# Polyarticular Septic Arthritis in an Immunocompetent Adult: A Case Report and Review of the Literature

**DOI:** 10.1155/2015/602137

**Published:** 2015-06-04

**Authors:** Annelise Miller, Fahad Abduljabbar, Peter Jarzem

**Affiliations:** ^1^McGill Scoliosis & Spine Centre, McGill University Health Centre, Montreal, QC, Canada; ^2^Department of Orthopedic Surgery, King Abdulaziz University, Jeddah, Saudi Arabia

## Abstract

Septic arthritis is a clinical emergency requiring prompt diagnosis and treatment to avoid significant morbidity and mortality. Polyarticular septic arthritis (PASA) accounts for 15% of all infectious arthritides and rarely occurs in immunocompetent adults. *Staphylococcus aureus* is the most commonly isolated organism, with infection primarily affecting knees, shoulders, elbows, and hips. The morbidity associated with PASA is very high, and mortality in treated cases of PASA may be as high as 50% of cases. We report a case of PASA with associated epidural abscess in a healthy adult male, who presented with complaints of arthralgia and limited range of motion of his left shoulder, wrist, and ankle. He also presented with low back pain and motor weakness associated with an epidural abscess spanning L2-S1, with multilevel vertebral osteomyelitis. Surgical washout of the affected joints as well as decompressive laminectomies was performed, and he received a standard course of intravenous antibiotics. *Staphylococcus aureus* was isolated from joint aspirations and from blood cultures. The patient had a full neurological and functional recovery postoperatively with no sequelae. To the best of our knowledge this is the only case report of *Staphylococcus aureus* PASA with concomitant epidural abscess in an immunocompetent adult.

## 1. Introduction

Septic arthritis is a clinical emergency, with significant morbidity and mortality especially with delayed diagnosis. Prompt diagnosis and intervention are required to prevent important functional complications of postinfectious joint destruction or, more seriously, septicemia, multiorgan failure, and even death. The incidence of septic arthritis is 2-3/100,000 [1]. It occurs predominantly as monoarticular septic arthritis (MASA), with the most commonly involved joints being the knee and hip, while polyarticular septic arthritis (PASA) accounts for 15% of cases, affecting primarily the knee, elbow, shoulder, and hip [2]. Dubost et al. have defined PASA as the involvement of 2 or more joints excluding the sacroiliac joints [2].* Staphylococcus aureus* is the most common causative organism in nongonococcal native joint septic arthritis cases in Europe and North America [2, 3]. The literature reports a mortality rate of 11% for monoarticular septic arthritis rising to 50% for polyarticular involvement despite treatment [4]. The overwhelming majority of cases of PASA are in patients with underlying rheumatological disease, immune compromise, malignancy, or chronic comorbid illness such as diabetes [2]. The occurrence of PASA in otherwise immunocompetent adults is exceedingly rare, with only a handful of reported cases [5–9]. In this paper, we report a rare case of polyarticular septic arthritis with epidural abscess in an otherwise healthy adult.

## 2. Case Report

A 56-year-old male construction worker presented to the emergency department with a two-week history of low back pain followed by left shoulder decreased range of motion and left wrist and ankle pain, in addition to associated fever and chills for two days. One week prior to his presentation, the patient sought medical advice for his back pain at an outpatient clinic; he was given a primary diagnosis of mechanical low back pain and his primary care physician referred him to physiotherapy. The patient had no history of a recent upper respiratory tract, gastrointestinal tract, or urinary tract infection. There was no history of recent travel, steroid use, or intravenous drug abuse. He denied alcohol abuse and smoking. Past medical history revealed dyslipidemia, as well as a previously drained perianal abscess two years before which was complicated by a fistula that was treated surgically one year prior to presentation.

Upon the patient's arrival to the emergency department, a temperature of 38.8°C was recorded and he was hemodynamically stable. Physical examination demonstrated low back tenderness and left shoulder swelling with limited and painful range of motion. His left wrist and ankle exam also showed decreased range of motion secondary to pain with moderate effusion. Cardiac examination did not reveal any murmurs. The neurological examination showed intact cranial nerves. His upper extremity neurological exam was unremarkable, but his lower extremity examination showed weakness of his right hip flexors and right knee extensors in comparison to the contralateral side (4/5). He had normal sensation and his biceps, triceps, and ankle reflexes were all normal.

No other foci of infection upon physical examination were noted.

Laboratory data revealed an elevated erythrocyte sedimentation rate (76 mm), an elevated C-reactive protein (420 mg/dL), and a white blood cell count of 17,000/mm^3^ (80% segmented neutrophils). Aspiration of the left shoulder returned purulent frank pus containing 148,000 leukocytes/mm^3^ with 80% neutrophils, and Gram stains showed Gram-positive cocci. Hepatitis and human immunodeficiency virus (HIV) serologies were negative both initially and upon repeat testing. Plain radiographs of the affected joints and lower spine did not show any pathology, and chest radiograph and abdominal CT scan were within normal limits. Magnetic Resonance Imaging (MRI) of his spine demonstrated a multiloculated epidural abscess spanning L2-S1 with subchondral bone marrow changes noted at the L2-L3 vertebrae with enhancement. It was noted to cause mass effect on the thecal sac with a moderate to severe central canal stenosis, worse at L3. Echocardiogram did not show any valvular vegetation.

The diagnoses of bacteremia, epidural abscess, and polyarticular septic arthritis were suggested; the patient was started on empirical intravenous antibiotic therapy using piperacillin-tazobactam and vancomycin. Surgical intervention included the following: epidural abscess evacuation, decompressive laminectomies, and left shoulder arthroscopic irrigation and debridement. Intraoperative aspiration of left ankle and wrist showed turbid looking synovial fluid, which was sent for cultures and sensitivity, after which the joints were also irrigated. The aspirated fluid was negative for crystals, and final synovial fluid, urine, and blood cultures were positive for* Staphylococcus aureus*. Based on these results the empirical intravenous antibiotics were changed to intravenous cloxacillin. Postoperatively, the patient regained full range of motion of his left shoulder, wrist, and ankle. His low back pain improved dramatically, and his L2-L3 weakness on the right side recovered. The patient was discharged home in a stable condition with a peripherally inserted central catheter to receive cloxacillin for 6 weeks.

## 3. Discussion

Polyarticular septic arthritis (PASA) in immunocompetent patients is poorly documented in the peer-reviewed literature; retrospective case studies suggest that PASA only accounts for 15% of all cases of infectious arthritis [2]. Risk factors predisposing to this condition include arthritic disorders, chronic systemic diseases, associated infections (including human immunodeficiency virus), malignancies, drug-induced immunosuppression, and intravenous drug abuse [8]. Unsurprisingly, there is a very strong association of septic arthritis in patients with rheumatoid arthritis [10]. Poor prognostic factors in PASA included advanced age (>50), staphylococcal infection, underlying rheumatoid arthritis, immunosuppression [2], and delayed diagnosis [7]. In 1993, Dubost et al. looked at a series of 25 cases of polyarticular septic arthritis. They found that 13 (52%) of the patients had underlying rheumatoid disease, 9 (36%) were known to be immunocompromised, and only 3 patients (12%) were immunocompetent. Only 5 other reports are published regarding such cases [5–9], and the organisms isolated varied greatly, including* Staphylococcus aureus* [8],* Streptococcus pyogenes* [5],* Streptococcus pneumoniae* [7], group A beta-hemolytic* Streptococcus* [6], and* Haemophilus influenzae* [9]. We found only one case report of PASA associated with epidural abscess caused by* Streptococcus pneumoniae* in an immunocompetent adult [7]. To our knowledge, there are no reported cases of PASA and epidural abscess due to* Staphylococcus aureus* in the literature.

Spinal epidural abscesses (SEA) “result from purulent material collecting between the spinal dural covering and osseous-ligamentous structures of the spine” [11]. The classic clinical triad is back pain, fever, and neurologic deficit, although this is reportedly present in only a minority of patients [12]. The condition is rare with incidences ranging from 0.2 to 1.2 cases per 10,000 hospital admissions per year, a number which has doubled in the last 2 decades due to an aging population, an increase in spinal instrumentation procedures, and a rise in the number of intravenous drug users (IVDUs) as well as patients with human immunodeficiency virus (HIV) infection [12, 13]. As in PASA, most patients with SEA have preexisting chronic disease; however other risk factors include existing spinal abnormality, past spinal intervention, and existing local or systemic infection [12, 14]. Up to 20% of patients have no risk factors for infection [11]. Most case reports cite a male to female risk ratio of 2 : 1, and there does not appear to be a specific age distribution, although case reports cluster in the 3rd to 7th decade range [13]. Contiguous spread accounts for about one-third of SEA cases, whereas hematogenous dissemination accounts for one-half of cases; in 20% of cases, the infectious source cannot be identified [11–13]. The lumbar spine is the most frequently affected region, followed by the thoracic, cervical, and sacral regions [11, 14].* Staphylococcus aureus* is the most commonly isolated organism (70% of cases), followed by streptococcal species (7%) and, uncommonly, Gram-negative cocci [12, 13]. Erythrocyte sedimentation rate is uniformly elevated in SEA; however, leukocytosis is less sensitive, with some case reports noting elevation in only 60–78% of cases [12, 13].

The entity of PASA is quite puzzling as these patients are frequently worked up for inflammatory conditions rather than a serious infectious process, more so given that twenty percent of patients are afebrile, and leukocytosis occurs in only 63% of cases [7]. The delay in the diagnosis of PASA may lead to substantial mortality (up to 50%), which is considerably higher than the inpatient mortality for myocardial infarction (10%) [3, 15]. Although some clinicians believe that concomitant septic and inflammatory arthritis is rare, this phenomenon has been documented [16]. The presence of a concomitant inflammatory arthritic condition such as rheumatoid arthritis cannot exclude PASA, as septic arthritides can frequently mimic RA flares. Care must be taken not to miss such cases. Our patient presents a challenging clinical picture due to the paucity of reported cases of PASA associated with epidural abscess in immunocompetent patients. Our literature search revealed only one other case in which both conditions occurred in an otherwise healthy adult; however, it was due to* Streptococcus pneumoniae* infection [7]. Fortunately, our patient had a favorable clinical outcome, having fully regained his range of motion postoperatively.

## 4. Conclusion

PASA is a puzzling clinical condition with a frequently delayed diagnosis. The possibility of polyarticular septic arthritis should be carefully considered in all cases of painful, swollen polyarthritis, even if patients are afebrile or have a normal white cell count. Whenever PASA is suspected, clinicians should have a low threshold to aspirate the affected joint to establish a diagnosis followed by early surgical drainage and intravenous antibiotic administration to avoid morbidity and mortality.
